# Postoperative granulomatous peritonitis mimicking abdominal tuberculosis

**DOI:** 10.1002/ccr3.1724

**Published:** 2018-07-25

**Authors:** Philipp Kasper, Katharina Pütz, Sarah Fünger, Isabelle Suárez, Norma Jung, Hakan Alakus, Christiane Bruns, Jan Rybniker

**Affiliations:** ^1^ Department of Gastroenterology and Hepatology University Hospital of Cologne Cologne Germany; ^2^ Institute of Pathology University Hospital of Cologne Cologne Germany; ^3^ Department I of Internal Medicine University Hospital of Cologne Cologne Germany; ^4^ German Center for Infection Research (DZIF), Partner Site Bonn‐Cologne Cologne Germany; ^5^ Department of General, Visceral and Cancer Surgery University Hospital of Cologne Cologne Germany; ^6^ Center for Molecular Medicine Cologne University of Cologne Cologne Germany

**Keywords:** abdominal tuberculosis, foreign material, granulomatous peritonitis, postoperative complication

## Abstract

Granulomatous peritonitis represents a rare postoperative complication that should be considered as important differential diagnosis in all patients who present to the hospital with abdominal pain, abdominal tenderness, and fever after abdominal surgery. Clinical distinction from abdominal tuberculosis remains a diagnostic challenge and requires thorough histopathological and microbiological examination.

## 1 INTRODUCTION

A 40‐year‐old woman presented to the hospital with right upper quadrant abdominal pain, flank pain, and fever. Seven weeks before this presentation, an emergency laparotomy had been performed in the Ukraine because of spontaneous liver capsule rupture of a so far unknown liver tumor. During the diagnostic workup, a re‐laparotomy for evaluation of the liver tumor was performed. Intraoperatively, multiple abscesses and peritoneal purulent nodules were detected within the peritoneal cavity. Frozen section analysis of taken biopsies revealed epithelioid cell granulomas with central caseous necrosis. Due to the clinical and histological suspicion of tuberculous peritonitis, the operation was terminated prematurely. Follow‐up analysis excluded abdominal tuberculosis using microbiological, histopathological, and molecular biological methods. However, subsequent extensive histological analysis revealed multiple phagocytosed cellulose fibers and other foreign material within the granulomas. Based on these results and the clinical course of the patient, a postoperative granulomatosis peritonitis induced by exposure to foreign material of the initial operation in the Ukraine was diagnosed.

Granulomatous peritonitis represents a rare postoperative complication that may occur after extensive abdominal operations and is often associated with surgery performed under emergency conditions. In such a situation, granulomatous peritonitis can be caused by foreign material contamination and can be triggered, eg, by surgical glove powder, foreign particles, or undigested particles from accidental intestinal perforation or cotton lint from disposable surgical drapes and laparotomy pads.

Clinical symptoms of postoperative granulomatous peritonitis are heterogeneous and vary from abdominal tenderness, intestinal obstruction, fever, ascites to mild and nonspecific abdominal pain.^1^, ^2^ Due to its diverse clinical presentation, the diagnosis of postoperative granulomatous peritonitis is a challenge for clinicians.

Abdominal tuberculosis, which is the sixth most frequent form of extra‐pulmonary tuberculosis, represents the most important differential diagnosis of postoperative granulomatous peritonitis.^1^, ^2^, ^3^, ^4^

The clinical, macroscopic, and microscopic characteristics of both entities are very similar. In addition, tuberculous peritonitis is equally difficult to diagnose primarily due to its insidious onset, due to its variability in symptoms, and due to a lack of easily applicable diagnostic biomarkers. However, pathophysiology as well as treatment differs fundamentally.

Based on these facts, this case report intends to highlight the important differences of both diseases in order to help clinicians to determine the right diagnosis and initiate the correct treatment.

## 2 CLINICAL HISTORY/EXAMINATION

A 40‐year‐old woman presented to the hospital with abdominal pain in the right upper quadrant right, flank pain, and fever. She denied dysuria, polyuria, haematuria, nausea, or vomiting.

Seven weeks earlier, an emergency laparotomy had been performed in the Ukraine because of a spontaneous liver capsule rupture of a liver tumor, which was unknown before. During the emergency operation, multifocal disseminated liver lesions were detected. Taken biopsies, considering the circumstances of reduced quality, were analyzed histologically, and a hepatocellular carcinoma (HCC) was initially suspected.

After she had an uneventful postoperative course, she was discharged from the hospital in the Ukraine. For re‐evaluation of the histological samples and oncological treatment, she traveled to Germany.

A few days after she had arrived, she appeared to our emergency department, presenting the above‐mentioned symptoms.

## 3 INVESTIGATIONS AND TREATMENT

At physical examination, she was awake, alert, and interacting appropriately. The cardiac examination was without pathological findings, and lungs were clear with breath sounds bilaterally. Abdominal examination revealed right upper quadrant abdominal tenderness and right flank tenderness.

The vital signs were as follows: systolic/diastolic blood pressure, 139/102 mm Hg: heart rate, 96 bpm; respiratory rate, 22 breaths/min; oxygen saturation (SpO2), 98% on room air; and body temperature, 38.2°C.

Laboratory tests revealed increased C‐reactive protein (CRP) level of 46.9 mg/dL (normal range: <5.0 mg/dL), a normocytic hypochromic anemia with a hemoglobin level of 9.8 g/dL (normal range: 12‐16 g/dL), hepatocellular injury with slightly elevated aspartate‐aminotransferase (AST) level of 40 U/L (normal range: <35 U/L), cholestasis with moderately increased alkaline phosphatase (AP) level of 165 U/L (normal range: 35‐105 U/L) and moderately elevated gamma‐glutamyl transferase (GGT) level of 101 U/L (normal range: <40 U/L) as well as markedly increased levels of lactate dehydrogenase (LDH) of 751 U/L (normal range: <250 U/L). Urine analysis revealed no features of urinary tract infection.

Abdominal ultrasound revealed a large, solid, exophytic tumor with irregular margins, mixed echogenicity occupying the main part of the right liver lobe and displacing the right kidney.

**Figure 1:**
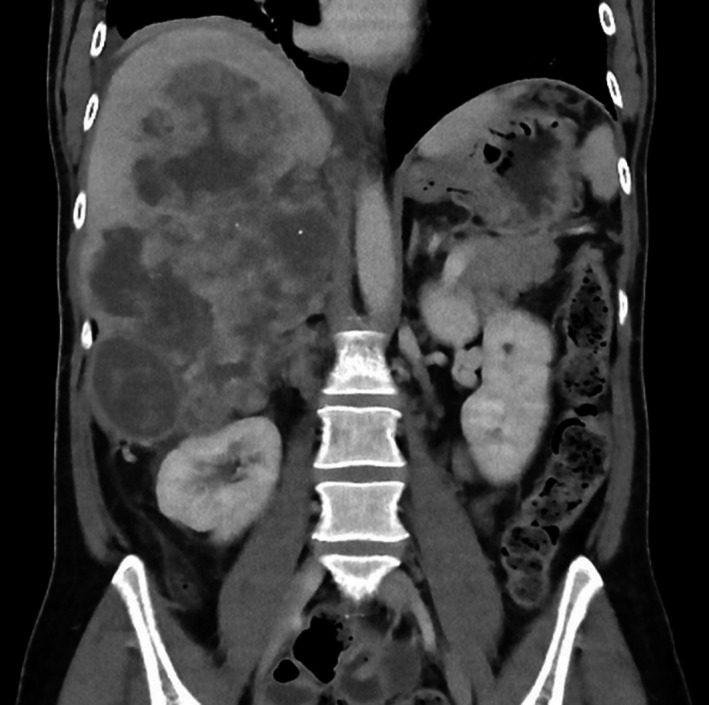
Abdominal CT scan. An axial contrast‐enhanced computed tomography (CT) image of the abdomen shows an inhomogeneous, large, nonenhanced hypodense lesion measuring 13.6 × 11.6 × 20 cm, occupying most of the right liver with exophytic components encroaching the upper right suprarenal region and displacing the right kidney inferiorly

Abdominal computed tomography (CT) with contrast confirmed the ultrasound results and showed an inhomogeneous, large, nonenhanced hypodense lesion measuring 13.6 × 11.6 × 20 cm, occupying most of the right liver with exophytic components encroaching the upper right suprarenal region and displacing the right kidney inferiorly. Furthermore, multiple enlarged abnormal para‐aortic and para‐caval lymph nodes were detected (Figure 1).

To re‐evaluate the liver tumor, a re‐laparotomy was performed.

At laparotomy, multiple extensive adhesions between intestinal loops and the anterior abdominal wall were detected. During adhesiolysis, multiple intestinal loops, especially the transverse colon, were found to be studded with purulent nodular lesions. Local excision biopsies from these lesions were taken, and intraoperative frozen section analysis was immediately performed.

**Figure 2:**
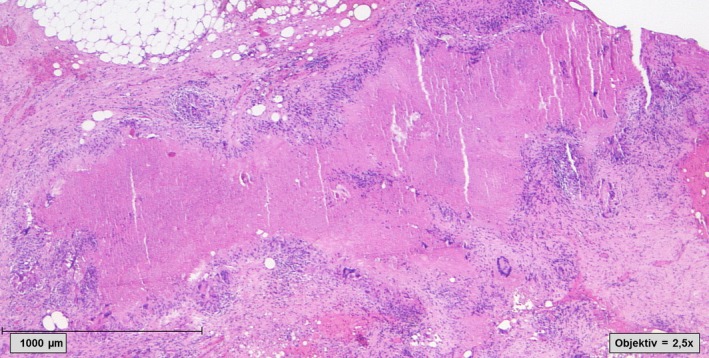
Histological finding of the peritoneal biopsies. Granulomatous inflammation with caseating necrosis within an area of fibrosis (Hematoxylin and Eosin staining, 2.5× magnification)

The histopathological analysis revealed granulomatous inflammation with epithelioid cell granulomas with central caseous necrosis (Figure 2). However, no malignant cells were detected.

Because of the appearance of miliary purulent lesions throughout the peritoneal cavity with caseating granuloma on histology, a tuberculous peritonitis was assumed and the operation was terminated prematurely.

However, during the following diagnostic workup, no acid‐fast bacilli were detected with Ziehl‐Neelsen staining of the intestinal tissue samples and polymerase chain reaction (PCR) targeting *M. tuberculosis* DNA was negative in all intestinal samples. Culture for *M. tuberculosis* remained sterile after 8 weeks of incubation. An interferon‐γ release assay (QuantiFERON TB gold test) was also negative. Because all diagnostic tests for *M. tuberculosis* from intestinal tissue samples were negative, the diagnosis of abdominal tuberculosis was discarded.

Extensive histological analysis of the tissue samples revealed an unexpected result.

**Figure 3:**
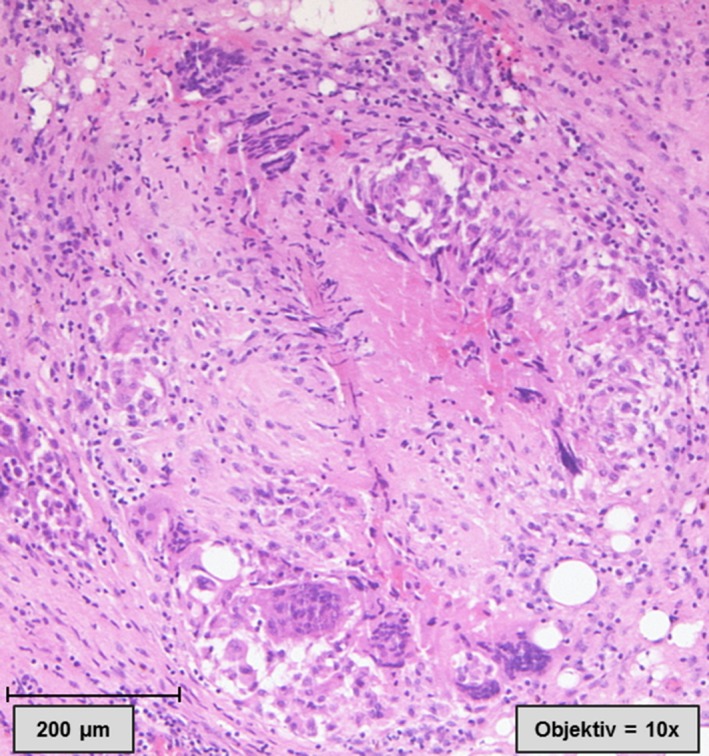
Histological finding of the peritoneal biopsies, showing an epithelioid granuloma with central necrotic regions. The granuloma is surrounded by a diffuse inflammatory cell infiltration, consisting of lymphocytes, neutrophils, plasma cells, foamy macrophages, and some multinucleate giant cells. Hematoxylin and Eosin staining, 10× magnification)

**Figure 4:**
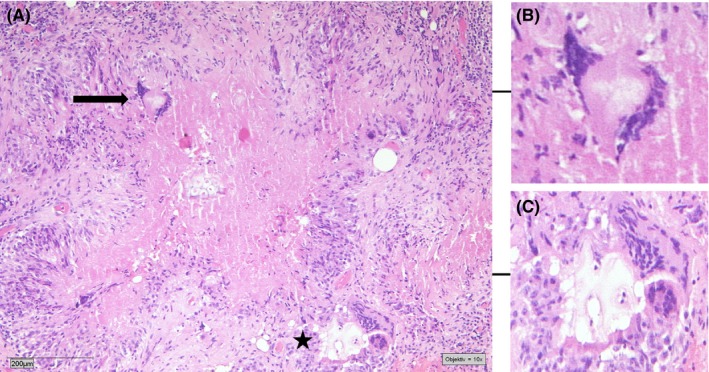
Histological finding of the peritoneal biopsies, showing focal fibrosis areas with local hemorrhages and epithelioid granulomas with central necrotic regions (A). Some giant cells contain phagocytosed foreign material (black arrow, B). The granuloma is surrounded by a diffuse inflammatory cell infiltration, consisting of lymphocytes, neutrophils, plasma cells, macrophages, and some multinucleate giant cells surrounding foreign material (black star, C). (Hematoxylin and Eosin staining, 10× magnification)

The taken biopsies of the subcutaneous tissue and the peritoneum showed focal fibrosis areas with local hemorrhages. Furthermore, epithelioid granulomas with central necrotic regions and scattered hemorrhages were identified. The granulomas were surrounded by a diffuse inflammatory cell infiltration, consisting of lymphocytes, neutrophils, plasma cells, macrophages, and some multinucleate giant cells. Surprisingly, some of the giant cells contained phagocytosed foreign material, which was not birefringent in polarized light. Further amorphous foreign material was detected near the central necrotic areas, without association to the giant cells. Finally, the foreign material was identified as cellulose plant fibers (Figures 3 and 4).

Thus, the detected lesions closely resembled tuberculous lesions but were shown on microscopy to be granulomata containing cellulose fibers.

Based on these results, a postoperative granulomatosis peritonitis induced by contamination with foreign material during the initial operation in the Ukraine was diagnosed.

## 4 OUTCOME AND FOLLOW‐UP

After exclusion of abdominal tuberculosis, the patient recovered quickly without medical intervention and there were no postoperative complications. To definitely diagnose the liver tumor which was seen in initial CT scans and the emergency operation in the Ukraine, a CT‐guided needle biopsy of one of the suspect liver lesions in the right liver lobe was performed.

Histological analysis of this biopsy revealed adrenal cortical carcinoma cells. Based on this, the diagnosis of an adrenal cortical carcinoma with diffuse infiltration of the liver was established and the patient was discharged for chemotherapy in a specialized center.

## 5 DISCUSSION

Granulomatous peritonitis is commonly associated with infectious, malignant, or idiopathic inflammatory conditions, including sarcoidosis, granulomatosis with polyangiitis, lymphoproliferative disorders, and tuberculosis. In rare cases, granulomatous peritonitis can be caused by foreign material contamination during surgery.^1^, ^2^ Peritonitis can be triggered by surgical glove powder, undigested particles from accidental intestinal perforation, cotton lint from disposable surgical drapes, and laparotomy pads or chemical antiseptics, which are used for peritoneal lavage, and it is then called *postoperative* granulomatous peritonitis.^1^, ^5^, ^6^, ^7^, ^8^, ^9^

The true extent of postoperative granulomatous peritonitis is unknown.

Clinical symptoms of postoperative granulomatous peritonitis are highly variable and heterogeneous. Patients with granulomatous peritonitis may either present with abdominal pain, abdominal tenderness, ascites, ileus, abdominal distension, and lymphadenopathy or with unspecific signs such as fever and progressive malaise.^2^ Typically, the symptoms occur a few weeks after abdominal surgery.^1^, ^2^, ^6^, ^8^ In our case, the initial emergency operation had been performed 7 weeks before the clinical symptoms occurred.

Potential complications of postoperative granulomatous peritonitis are peritoneal adhesions with consecutive intestinal obstructions or intestinal fistula formation.^1^, ^10^

The pathophysiology of postoperative granulomatous peritonitis remains poorly understood. It is assumed that accidental intraperitoneal introduction of foreign material (eg, cellulose fibers after intestinal perforation or cornstarch from surgical glove powder) induces a delayed cell‐mediated hypersensitive inflammatory response with the formation of foreign body granulomas in susceptible individuals.^1^, ^2^, ^8^, ^11^, ^12^

Because of the variable clinical presentation, the diagnosis of postoperative granulomatous peritonitis represents a challenge for clinicians. In order to find the right diagnosis in due time, all patients, presenting to the hospital with unclear abdominal pain, abdominal tenderness, and fever, should be asked for abdominal surgery performed during the past weeks or months. Laboratory findings are often nonspecific. The blood samples could reveal leukocytosis with left shift and acidosis. Imaging tests, including a plain abdominal film, computerized tomography (CT), or an ultrasound examination, can be performed to detect perforations of the intestinal tract, ascites, abscess formation, or peritoneal thickening. If ascites fluid is detectable, a diagnostic paracentesis should be performed.

At explorative laparoscopy or laparotomy, the peritoneum and omentum are mostly studded with multiple small nodules.^1^, ^2^, ^8^, ^12^

Microscopic examination of the granulomas may reveal a wall of epithelioid histiocytes, enclosing either caseous material, masses of disintegrating nuclei, or foreign material particles like cellulose fiber material.^8^ Furthermore, multinucleate Langhans or foreign‐body‐typed giant cells containing foreign material particles, surrounded by eosinophils and lymphocytes, can be detected. Mononuclear macrophages and areas of interstitial focal fibrosis may also be traceable.^6^, ^8^, ^12^

The most important differential diagnosis to (foreign body) postoperative granulomatous peritonitis represents tuberculous peritonitis and peritoneal carcinomatosis.

Tuberculous peritonitis is frequently confused with postoperative granulomatous peritonitis as many clinical and histological features are almost identical.

Tuberculous peritonitis is equally difficult to diagnose primarily due to its insidious onset and variability in symptoms, and, like postoperative granulomatous peritonitis, tuberculous peritonitis may present with abdominal pain, abdominal tenderness, and fever.^3^, ^4^, ^13^, ^14^

On microscopy, tuberculous peritonitis is also characterized by epithelioid cell granulomas with central caseous necrosis.^3^, ^4^, ^13^, ^14^ Differentiation of the two disease entities requires thorough microbiological processing of the samples to exclude the presence of *M. tuberculosis* or other mycobacteria.

Risk groups for both diseases differ clearly.

Etiologically postoperative granulomatous peritonitis typically occur in patients with a medical history of surgery, whereas tuberculous peritonitis is common in those patients with an immunocompromised state, eg, due to end‐stage renal disease, old age, diabetes mellitus, or HIV infection.

In our case, the detected lesions closely resembled tuberculosis lesions, but were shown on microscopy to be granulomas containing foreign material—cellulose particles.

In the literature, a few cases of peritoneal granulomatous reaction to cellulose fibers are described.^6^, ^8^, ^9^, ^12^ They mostly followed bowel perforation and also clinically mimicked tuberculosis in some cases.^9^ Interestingly, the clinical and histopathological features as well as the timeline of the conditions we observed in our patient were similar to those described in other reports of this rare postoperative complication.

In our case, the origin of the cellulose fibers is not entirely clear. There are several possible sources of cellulose contamination in the emergency operation setting in the Ukraine: (a) cellulose fibers originating from disposable surgical fabrics like gowns, drapes, or laparotomy pads, which have been used during the emergency laparotomy and (b) cellulose fibers originating from digested chymus which was released from the intestinal lumen into the peritoneal cavity after injury of an intestinal loop.

## 6 CONCLUSION

In conclusion, postoperative granulomatous peritonitis represents a rare disease but should be considered as differential diagnosis in patients who present with abdominal pain, abdominal tenderness, and fever after abdominal surgery. Clinical distinction from abdominal tuberculosis, which represents the most important differential diagnosis, remains a diagnostic challenge. In most cases, the proper diagnosis can only be established by abdominal biopsy and thorough histopathological and microbiological examination of samples.

## CONFLICT OF INTEREST

None declared.

## AUTHORSHIP

PK: internist, wrote the manuscript, reviewed the literature, involved in the management of the patient. KP: pathologist, revised and reviewed the manuscript, created the histological images. HK and CB: surgeon, revised and reviewed the manuscript. NJ, SF, and IS: internist, involved in the management of the patient, reviewed the manuscript. JR: internist, revised and reviewed the manuscript, reviewed the literature, involved in patient management.
